# Editorial: Novel diagnostic and therapeutic strategies for retinal diseases, volume II

**DOI:** 10.3389/fmed.2023.1234119

**Published:** 2023-07-03

**Authors:** Dong Fang, Yun Wang, Hetian Lei, Shaochong Zhang

**Affiliations:** Shenzhen Eye Hospital, Shenzhen Eye Institute, Jinan University, Shenzhen, China

**Keywords:** retinal diseases, vascular endothelial growth factor, ischemic retinopathies, myopic maculopathy progression, diagnostic and therapeutic strategies

Retinopathies, including diabetic retinopathy (DR), proliferative vitreoretinopathy (PVR), macular degeneration, and retinitis pigmentosa (RP), can lead to blindness without timely treatment. Vascular endothelial growth factor (VEGF) has been identified as a key factor promoting vascular leakage and angiogenesis, making neutralization of VEGF the first-line therapy for ischemic retinopathies such as DR and retinal occlusions. However, not all patients with these retinopathies respond to anti-VEGF therapy, necessitating the exploration of novel treatments for these angiogenesis-related retinal diseases. Molecular genetics has not only helped in clarifying diagnoses but also enabled the treatment of inherited retinal diseases. This Research Topic aims to identify new therapeutic approaches for a variety of retinal diseases, including DR, retinal occlusions, retinal tear, retinal detachment, epiretinal membrane, macular hole, macular degeneration, and retinitis pigmentosa.

Liang et al. compared the efficacy and safety of intravitreal ranibizumab (IVR) monotherapy vs. intravitreal ranibizumab plus dexamethasone (IVR+DEX) implant for macular edema (ME) secondary to retinal vein occlusion (RVO). Their findings confirmed that treatment with IVR+DEX implant provided a significant improvement in best-corrected visual acuity (BCVA) and reduction in central foveal thickness (CFT) in CRVO-ME.

Zhang et al. investigated and compared the concentration of cytokines in the aqueous humor (AH) and vitreous mass in high myopias (HM) with and without myopic choroidal neovascularization (mCNV). Their study confirmed the increased secretion of VEGF in eyes with mCNV, suggesting the involvement of inflammatory cytokines in the development of both HM and mCNV. The upregulation of inflammatory cytokines may precede the upregulation of VEGF. The vitreous could potentially serve as a more reliable biomarker in eyes with longer axial length.

Mao et al. studied the retinal vascular morphological characteristics in high myopia patients with varying degrees of severity using Ultra-Wide Field Images and artificial intelligence with a transfer learning system. Their analysis using the RU-net and transfer learning technology demonstrated high accuracy (98.24%) in quantitatively analyzing vascular morphological characteristics in Ultra-wide field images. They observed a decrease in vessel angle, vessel density, and vascular branches with the increase in myopic maculopathy severity and elongation of the eyeball. Patients with myopic CNV exhibited higher vessel density and more vascular branches.

Fomo et al. demonstrated that a synthetic antibody-derived immunopeptide could provide neuroprotection in glaucoma through its molecular interaction with retinal protein Histone H3.1.

Wu et al. showed that fullerenol, a potent nanomaterial, protects retinal pigment epithelial (RPE) cells from reactive oxygen species (ROS) attack, offering a promising approach for the prevention of age-related macular degeneration (AMD).

Seah et al. reviewed the role of polymeric biomaterials in the treatment of posterior segment diseases. These biomaterials can be engineered to function as endotamponade agents and prevent intraocular scarring in retinal detachment repair surgeries. They can also serve as drug delivery platforms for the treatment of retinal diseases and provide scaffolds for cellular products. Additionally, they offer non-viral gene delivery solutions to the retina.

Ming and Qin performed a bibliometric and knowledge graph analysis to study the research status and trends in microperimetry in ophthalmology between 1992 and 2022. Their analysis provided valuable information for global researchers, facilitating future collaborations and tracking cutting-edge progress.

Collectively, this Research Topic of articles emphasizes the need for further exploration of novel diagnostic and therapeutic approaches for a variety of retinal diseases.

## Author contributions

DF and YW drafted the manuscript. HL and SZ critically proofread and edited the manuscript. All authors contributed to manuscript writing as well as revision and approved the submitted version.

